# Biomarkers expression in benign breast diseases and risk of subsequent breast cancer: a case–control study

**DOI:** 10.1002/cam4.1080

**Published:** 2017-05-04

**Authors:** Margarita Posso, Josep M. Corominas, Laia Serrano, Marta Román, Isabel Torá‐Rocamora, Laia Domingo, Anabel Romero, María Jesús Quintana, María Vernet‐Tomas, Marisa Baré, Carmen Vidal, Mar Sánchez, Francina Saladié, Carmen Natal, Joana Ferrer, Sònia Servitja, María Sala, Xavier Castells, the BELE Study Group, Andrea Burón, Ana Rodríguez‐Arana, Ana Romero, Mar Vernet, Xavier Andreu, Núria Milà, Laura López‐Vilaró, Ana Fernández‐Somoano, Jaume Galceran, Josep Alfons Espinàs

**Affiliations:** ^1^ Department of Epidemiology and EvaluationIMIM (Hospital del Mar Medical Research Institute)BarcelonaSpain; ^2^ Department of Clinical Epidemiology and Public HealthHospital de la Santa Creu i Sant Pau (IIB Sant Pau)BarcelonaSpain; ^3^ Pathology DepartmentIMIM (Hospital del Mar Medical Research Institute)BarcelonaSpain; ^4^ Research Network on Health Services in Chronic Diseases (REDISSEC)BarcelonaSpain; ^5^ Agency for Health Quality and Assessment of Catalonia (AQuAS)BarcelonaSpain; ^6^ CIBER of Epidemiology and Public Health (CIBERESP)BarcelonaSpain; ^7^ Obstetrics and Gynecology DepartmentIMIM (Hospital del Mar Medical Research Institute)BarcelonaSpain; ^8^ Clinical Epidemiology and Cancer ScreeningParc Taulí University HospitalBarcelonaSpain; ^9^ Cancer Prevention and Control ProgramCatalan Institute of OncologyBarcelonaSpain; ^10^ Direction General of Public HealthDepartment of HealthGovernment of CantabriaSantanderSpain; ^11^ Breast Cancer Screening Program of TarragonaThe Foundation League for the Research and Prevention of CancerTarragonaSpain; ^12^ Principality of Asturias Breast Cancer Screening ProgramPrincipality of AsturiasOviedoSpain; ^13^ Radiology DepartmentHospital de Santa CaterinaGironaSpain; ^14^ Oncology DepartmentIMIM (Hospital del Mar Medical Research Institute)BarcelonaSpain

**Keywords:** Benign breast disease, biomarkers, breast cancer, early detection, screening

## Abstract

Women with benign breast diseases (BBD) have a high risk of breast cancer. However, no biomarkers have been clearly established to predict cancer in these women. Our aim was to explore whether estrogen receptor (ER), progesterone receptor (PR), and Ki67 expression stratify risk of breast cancer in screened women with BBD. We conducted a nested case–control study. Women with breast cancer and prior BBDs (86 cases) were matched to women with prior BBDs who were free from breast cancer (172 controls). The matching factors were age at BBD diagnosis, type of BBD, and follow‐up time since BBD diagnosis. ER, PR, and Ki67 expression were obtained from BBDs’ specimens. Conditional logistic regression was used to estimate odds ratios (ORs), and 95% confidence intervals (CIs) of breast cancer risk according to ER, PR, and Ki67 expression. Women with >90% of ER expression had a higher risk of breast cancer (OR = 2.63; 95% CI: 1.26–5.51) than women with ≤70% of ER expression. Similarly, women with >80% of PR expression had a higher risk of breast cancer (OR = 2.22; 95% CI: 1.15–4.27) than women with ≤40% of PR expression. Women with proliferative disease and ≥1% of Ki67 expression had a nonsignificantly increased risk of breast cancer (OR = 1.16; 95% CI: 0.46–2.90) than women with <1% of Ki67 expression. A high expression of ER and PR in BBD is associated with an increased risk of subsequent breast cancer. In proliferative disease, high Ki67 expression may also have an increased risk. This information is helpful to better characterize BBD and is one more step toward personalizing the clinical management of these women.

## Introduction

Benign breast diseases (BBD) are associated with an increased risk of subsequent breast cancer 1, 2. The type of histological abnormality of BBD stratifies this risk. Although proliferative disease with and without atypia could be associated with a two‐ to fourfold increased risk of developing breast cancer, nonproliferative diseases have a minimal increased risk of breast cancer 1.

The introduction of mammographic screening has led to a rise in the detection of BBD 2, 3, 4. There are a number of mammographic features which, when considered suspicious, can lead to further diagnostic tests reaching a nonmalignant confirmation 5. We have previously described that approximately 1.8% of screened women were found to have a BBD in our Spanish cohort 6. Although it is possible that intensive screening can enhance the benefits for women with BBD, in fact, the majority of these women are still following routine screening recommendations 2, 4.

In order to better understand the biological characteristics of BBD, a few authors have focused on estrogen receptor (ER) 7, 8, progesterone receptor (PR) 9, and Ki67 7, 10, 11 as biomarkers expressed in benign lesions that are not in proximity to concomitant breast cancer. The use of these biomarkers in the common clinical practice was born out by the fact that they have prognostic and response to treatment significance in women with breast cancer tumors 12, 13. In women with BBD, however, the study of the expression of ER, PR, and Ki67 as predictors of subsequent breast cancer has shown inconsistent results 7, 8, 9, 10, 11. Although some studies reported an increased risk of subsequent cancer in women with BBD that had high ER, PR, or Ki67 expression 7, 8, 9, 11, others reported different or contrary results 7, 14, 15.

To date, no biomarkers have been clearly established to predict cancer in women diagnosed with BBD. Moreover, as far as we know, studies that assessed these biomarkers have not been performed in breast cancer screening programs. Ideally, the identification of these biomarkers could improve both our ability to stratify an individual's risk for breast cancer and lead to more accurate follow‐up or screening strategies. Therefore, the aim of our study was to explore whether ER and PR expression stratify risk of breast cancer in screened women with BBD. We also investigated the Ki67 expression in women with proliferative disease.

## Material and Methods

### Setting

The Spanish breast cancer screening program adheres to the recommendations of the European Guidelines for quality assurance in breast cancer screening and diagnosis 16. Characteristics of the Spanish program have been previously described 6. Briefly, women aged 50–69 years are routinely invited for a biennial screening. Biennial screening consists of two mammography views of each breast, a mediolateral oblique, and a cranio‐caudal view. If the mammogram is negative, the woman is invited for further mammography screening in 2 years. On the contrary, if suspicious mammographic findings are identified, the woman is recalled for further assessment to rule out malignancy. Further assessment often includes imaging procedures (i.e., additional mammography, ultrasonography, and magnetic resonance imaging) and/or biopsies (i.e., fine‐needle aspiration, core needle biopsy, and open biopsy).

### Study design

This study was designed as a nested case–control study among the subset of women with pathology‐confirmed BBD within our Spanish screening cohort. The initial cohort subset included 10,262 women with BBD diagnosed in a breast cancer screening program between 1994 and 2011. Characteristics of our cohort have been previously reported 6. Biopsies with indeterminate histological classification, for example, “negative for malignant cells” (*N* = 4251), were excluded from the analysis because they could not be classified in any of the BBD subtypes. Most of these biopsies with indeterminate classification came from fine‐needle aspiration cytology. Therefore, 6011 women with BBD were followed up until December 31, 2015. Each BBD was classified into one of the following types: (1) nonproliferative disease; (2) proliferative disease without atypia; and (3) proliferative disease with atypia 17, 18, 19.

Cases and controls were selected from the study cohort subset. Cases were women with BBD who developed breast cancer during follow‐up, whereas controls were women with BBD who remained cancer‐free at least longer than the time to cancer in matched cases. From the 6011 women with BBD, 201 (3.3%) developed breast cancer during follow‐up and were considered cases. We excluded 114 cases because they did not have sufficient tissue to assess biomarker's expression in the BBD sample and one case because it had evidence of mucinous carcinoma in the BBD sample. Thus, 86 cases with sufficient tissue to assess biomarkers’ expression were included in the analysis. We randomly selected two controls per each case. Cases and controls were matched by age at the time of BBD diagnosis, type of BBD (nonproliferative disease, proliferative disease without atypia, or proliferative disease with atypia), and follow‐up time between BBD diagnosis and end of follow‐up. The sample size for the analysis included 258 women, 86 cases matched to 172 controls.

Both invasive and in situ carcinomas detected at regular screening or until the end of follow‐up were analyzed. Age at BBD diagnosis, diagnosis of BBD, follow‐up time between BBD diagnosis and the end of follow‐up, and breast cancer events were retrieved from the breast cancer screening program database and hospital records. All protocol procedures and methods of ascertainment of BBD and breast cancer diagnosis were reviewed and approved by the ethical committees at all participating institutions.

### Pathology biopsies (ER, PR, Ki67 expression measurement)

Two pathologists independently reviewed the hematoxylin and eosin (H&E) slides from the biopsy blocks for the BBDs. The pathologists were blinded to each biopsy's case or control status. They completed detailed worksheets on the ER, PR, and Ki67 expressions when applied, and the subtype of BBD lesion (i.e., nonproliferative, proliferative without atypia, and proliferative with atypia) 17, 18, 19. In the case of disagreement between pathologists, the quantitation was determined by consensus. If the woman had more than one biopsy during the study period, we used findings from the earliest biopsy performed. Individual tissue samples that exhibited more than one pathology diagnosis were classified by the most severe pathology finding.

Samples were fixed in 4% buffered formalin and embedded in paraffin. Four‐micrometer sections of formalin‐fixed, paraffin‐embedded samples were deparaffinized and rehydrated. Staining H&E control and immunohistochemistry staining were performed for detecting ER, PR, and Ki67 expressions. Suitable positive tissue controls were used as quality control. Immunohistochemistry staining was performed using the Benchmark XT (VENTANA Roche).

To study ER and PR, we used the retrieval antigen protocol, endogenous peroxidase inhibition, detection system, DAB, and Hematoxylin from the UltraView system (VENTANA Roche). The primary antibodies used for ER and PR detection were Rabbit anti‐human estrogen receptor alpha (clone SP1) prediluted (VENTANA Roche), and rabbit anti‐human progesterone receptor (clone 1E2) prediluted (VENTANA Roche) and incubated for 20 min at 37°C.

To study Ki67, we used the retrieval antigen protocol, endogenous peroxidase inhibition, detection system, DAB, and Hematoxylin from the OptiView system (VENTANA Roche). The primary antibody used for Ki67 expression detection was rabbit anti‐human Ki67 (clon 30‐9) prediluted (VENTANA Roche) and incubated for 12 min at 37°C.

The percentage of ER, PR, and Ki67 staining was scored based on the number of positively stained nucleus per 100 studied cells observed with microscopic examination. At first, the entire sample was visualized in order to find the areas corresponding to the BBD lesion. If there were more than one BBD lesion, we evaluated the more severe one (i.e., proliferative disease with atypia followed by proliferative disease without atypia and by nonproliferative disease). In the case of proliferative disease, we evaluated the biomarker's expression in the areas with the highest proliferative activity. In the case of nonproliferative disease, we evaluated the biomarker's expression in the entire area of the BBD lesion. We did not evaluate the background normal lobules.

Pathologic classification of cancer such us invasive carcinoma and carcinoma in situ were ascertained from hospital records.

### Statistical analysis

The percentage of ER‐, PR‐, and Ki67‐positive cells in each sample was obtained by the pathologists’ microscopic assessment. We attempted to categorize the distribution of the ER and PR percentages into tertiles and we obtained the following groups: For ER (1) low expression: 0–70%; (2) moderate expression: 71–90%; and (3) high expression: >90%. For PR (1) low expression: 0–40%; (2) moderate expression: 41–80%; and (3) high expression: >80%. Regarding women with proliferative disease, Ki67 was categorized into two groups: (1) low expression: 0% to <1%; and (2) high expression: ≥1%. We calculated the median and range for continuous variables (age at BBD diagnosis, follow‐up time between BBD diagnosis and the end of follow‐up) and t‐tests were used for comparisons. Chi‐square tests were used to compare the distribution of ER, PR, and Ki67 expression across types of BBD and case–control status. Conditional logistic regression was used to estimate the odds ratios (ORs) and 95% confidence intervals (CIs) of breast cancer risk according to ER, PR, and Ki67 expression. We classified women according to the type of BBD and calculated ORs per each group. *P*‐Value < 0.05 was considered statistically significant. Statistical analyses were performed using SPSS (v.19).

## Results

### Patients

Of the 201 subsequent breast cancer cases diagnosed from 1994 to 2015 in the study population, 86 had a previous BBD sample with sufficient tissue to assess ER and PR expression and were included in the analysis. We randomly selected two controls per each case. Thus, the sample size for the analysis included 258 women, 86 cases matched to 172 controls. Of the 258 women with BBD, 65.1% (*N* = 168) were classified as nonproliferative disease, 30.2% (*N* = 78) as proliferative disease without atypia, and 4.7% (*N* = 12) as proliferative disease with atypia. Overall, the mean age at BBD diagnosis was 56.4 years (56.3 and 56.5 years for cases and controls, respectively). Median time to cancer diagnosis among cases was 71.4 months (range, 18–193 months), and median length of cancer‐free follow‐up among controls was very similar (69.2 months, range, 12–183 months; *P* = 0.69) (Table 1). Breast cancer cases were screen‐detected in 78% of all cases (*N* = 67/86), whereas the 22% (*N* = 19/86) was identified out of the regular screening. Seventy‐seven percent (*N* = 66/86) of cases were invasive carcinomas, 14% (*N* = 12/86) were in situ, and the presence of invasion was unknown in 9% (*N* = 8/86) of cases.

**Table 1:** Characteristics of the nested case‐control sample

	Total (*N* = 258)		Case (*N* = 86)		Control (*N* = 172)		*P*‐value
Benign breast disease (BBD)							
Nonproliferative (*N*, %)	168	65.1%	56	65.1%	112	65.1%	1.00^b^
Proliferative without atypia (*N*, %)	78	30.2%	26	30.2%	52	30.2%	
Proliferative with atypia (*N*, %)	12	4.7%	4	4.7%	8	4.7%	
Age at BBD diagnosis, years							
Mean, SD	56.4	0.3	56.3	0.5	56.5	0.4	0.70^c^
Median (Range)	56	(49–68)	55	(50–68)	56	(49–67)	
Age groups							
50–54 (*N*, %)	103	39.9%	36	41.9%	67	39.0%	0.50^b^
55–59 (*N*, %)	78	30.2%	29	33.7%	49	28.5%	
60–64 (*N*, %)	66	25.6%	19	22.1%	47	27.3%	
65–68 (*N*, %)	11	4.3%	2	2.3%	9	5.2%	
Years since BBD^a^, months							
Mean, SD	70.0	2.5	71.4	4.6	69.2	3.0	0.69^c^
Median (Range)	63.5	(12–193)	67.0	(18–193)	62.0	(12–183)	

a Follow‐up time between year of BBD diagnosis and the end of follow‐up.

b Chi‐squared test comparing distributions of characteristics across case‐control status.

c Two‐sample t‐tests comparing means across case‐control status.

### ER expression

**Figure 1:**
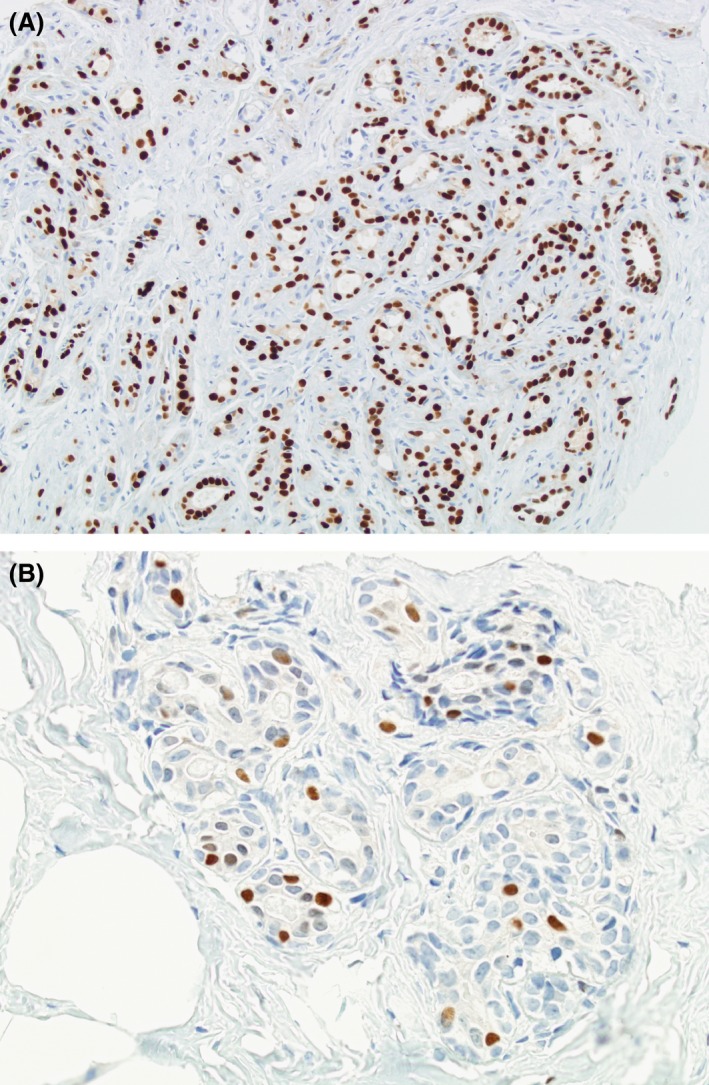
Estrogen receptor expression. (A) High expression (>90%). (B) Low expression (0–70%).

Nearly all BBD samples (99.2%; *N* = 256/258) showed ≥1% of ER immunostaining expression. The highest ER expression was 98% (Fig. 1A), observed in 30.6% (*N* = 79/258) of all BBD samples. The ER expression mean scores were 83% and 74% (*P* < 0.01) in cases and controls, respectively. A higher proportion of BBD samples with >90% positively stained cells were observed in cases than in controls (Table 2). Compared to women with low ER expression (≤70%), women with moderate (71% to 90%) and high expression (>90%) had an elevated risk of breast cancer (OR = 1.98; 95% CI: 1.01–3.89 and OR = 2.63; 95% CI: 1.26–5.51, respectively) (Table 3). Restricting analyses to nonproliferative disease and to proliferative disease, we observed a similar tendency. For nonproliferative disease, ORs were nonsignificantly higher in women with moderate and high ER expression compared to women with low ER expression (OR = 1.85; 95% CI: 0.86–4.01 and OR = 2.42; 95% CI: 0.99–5.91, respectively). For proliferative disease, ORs were nonsignificantly higher in women with moderate and high ER expression compared to women with low ER expression (OR = 2.46; 95% CI: 0.59–10.29 and OR = 3.28; 95% CI: 0.82–13.13, respectively) (Table 3).

**Table 2:** Distribution of estrogen receptor (ER), progesterone receptor (PR), and Ki‐67 expression according to the case‐control status

	Total (*N* = 258)		Case (*N* = 86)		Control (*N* = 172)		*P*‐value^b^
	*N*	%	*N*	%	*N*	%	
ER expression							
≤70%	80	31.0	18	20.9	62	36.1	0.04
71–90%	98	38.0	35	40.7	63	36.6	
>90%	80	31.0	33	38.4	47	27.3	
PR expression							
≤40%	83	32.2	23	26.7	60	34.9	0.02
41–80%	83	32.2	22	25.6	61	35.5	
>80%	92	35.7	41	47.7	51	29.7	
Proliferative disease	Total (*N* = 90)		Case (*N* = 30)		Control (*N* = 60)		
Ki67 expression^a^							
0% to <1%	26	28.3	8	26.7	18	30.0	0.74
≥1%	64	71.7	22	73.3	42	70.0	

a Ki67 expression was evaluated only for proliferative disease.

b Chi‐squared test comparing distributions of characteristics across case‐control status.

**Table 3:** Odds ratios (OR) of subsequent breast cancer according to the percentage of estrogen receptor (ER), progesterone receptor (ER), and Ki67 expression

	All benign breast diseases^a^			Nonproliferative disease			Proliferative disease^b^		
	*N*	OR	(IC 95%)	*N*	OR	(IC 95%)	*N*	OR	(IC 95%)
ER expression									
≤70%	80	1		56	1		24	1	
71–90%	98	1.98	(1.01–3.89)	66	1.85	(0.86–4.01)	32	2.46	(0.59–10.29)
>90%	80	2.63	(1.26–5.51)	46	2.42	(0.99–5.91)	34	3.28	(0.82–13.13)
PR expression									
≤40%	83	1		60	1		23	1	
41–80%	83	0.87	(0.42–1.78)	56	0.68	(0.28–1.66)	27	1.43	(0.38–5.31)
>80%	92	2.22	(1.15–4.27)	52	2.48	(1.13–5.44)	40	1.81	(0.56–5.90)
Ki67 expression^c^									
0% to <1%	–	–	–	–	–	–	26	1	
≥1%	–	–	–	–	–	–	64	1.16	(0.46–2.90)

OR, Conditional logistic regression was used to estimate odds ratios, and 95% confidence intervals of breast cancer risk according to ER, PR, and Ki67 expression. IC 95%, 95% confidence interval.

a Women with nonproliferative disease, proliferative disease with atypia, and proliferative disease without atypia were included in the analyses.

b Women with proliferative disease with and without atypia were included in the analyses.

c Ki67 expression was evaluated only for proliferative disease.

### PR expression

**Figure 2:**
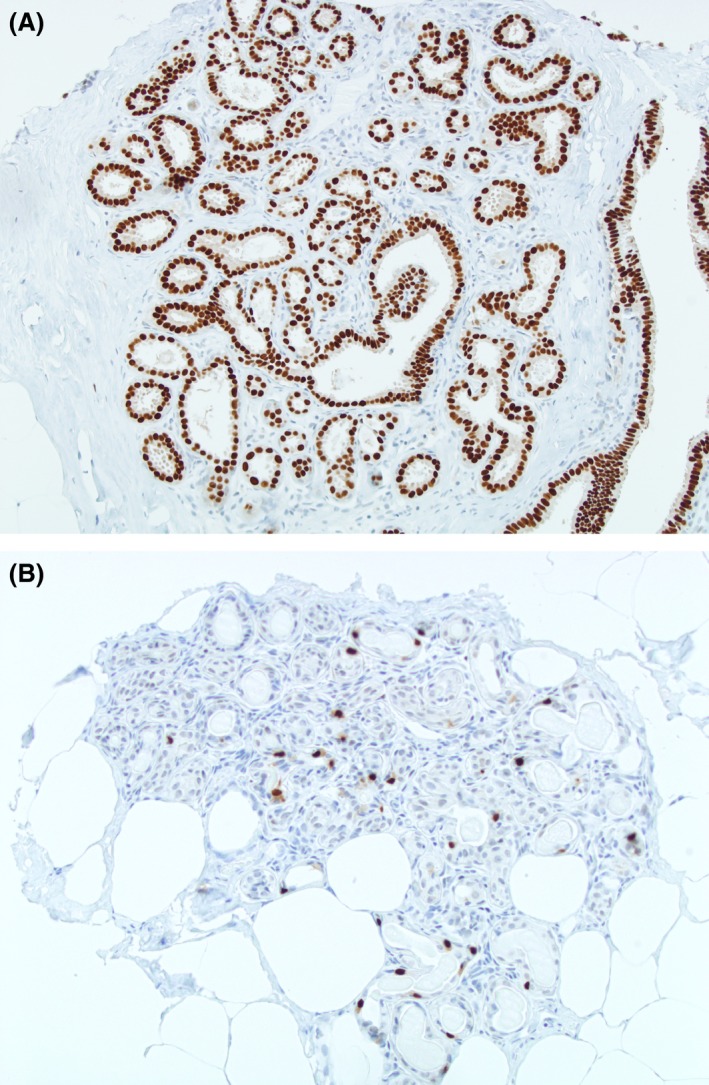
Progesterone receptor expression. (A) High expression (>80%). (B) Low expression (0–40%).

Two hundred and fifty‐two of 258 (97.7%) BBD samples showed ≥1% of PR immunostaining expression. The highest PR expression was 98% (Fig. 2A), observed in 12.4% (*N* = 32/258) of all BBD samples. The PR expression mean scores were 68% and 58% (*P* < 0.01) in cases and controls, respectively. A higher proportion of BBD samples with >80% positively stained cells were observed in cases than in controls (Table 2). Compared to women with low PR expression (≤40%), women with high PR expression (>80%) had an elevated risk of breast cancer (OR = 2.22; 95% CI: 0.15–4.27) (Table 3). Restricting analyses to nonproliferative disease and to proliferative disease, we observed a similar tendency. For nonproliferative disease, the OR was higher in women with high PR expression compared with women with low PR expression (OR = 2.48; 95% CI: 1.13–5.44). For proliferative disease, the OR was higher in women with high PR expression compared with women with low PR expression (OR = 1.81; 95% CI: 0.56–5.90) (Table 3).

### Ki67 expression in women with proliferative disease

**Figure 3:**
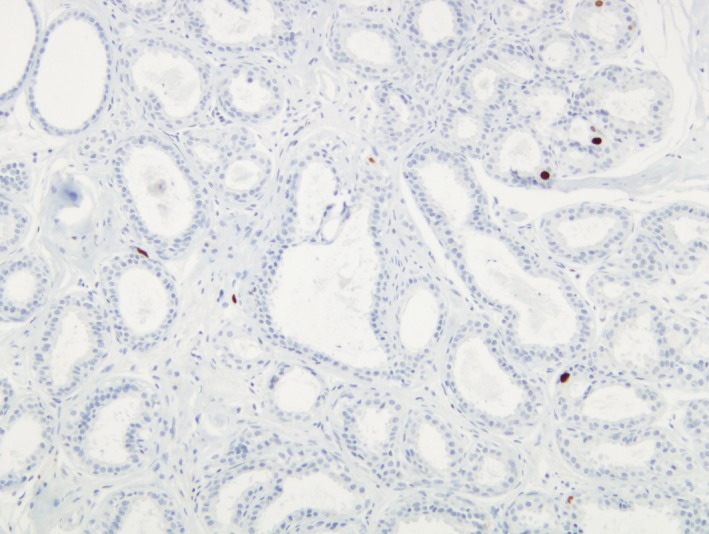
Ki67 < 1% of positive cells expression.

Almost all (98.9%; *N* = 89/90) proliferative disease samples showed some degree of Ki67 immunostaining expression; 28.9% (*N* = 26/90) of samples showed <1% of Ki67 expression (Fig. 3 and Table 2). The highest Ki67 expression was 10%, observed in one control. A similar proportion of BBD samples with ≥1% positively stained cells were observed in cases (73.3%; *N* = 22/30) than in controls (70%; *N* = 42/60; *P* = 0.11) (Table 2). Compared to women with <1% of Ki67 expression, women with ≥1% of Ki67 expression had a no significant increase in risk of breast cancer (OR = 1.16; 95% CI: 0.46–2.90) (Table 3).

## Discussion

In this case–control study, we assessed whether the expression of estrogen receptors (ER) and progesterone receptors (PR) stratifies the risk of subsequent cancer in women with benign breast diseases (BBD). We found both that women with high expression of ER or PR in BBD had an increased risk of subsequent breast cancer. This increased risk remains present when we analyzed women with nonproliferative disease. To the best of our knowledge, there is scarce evidence about the biomarkers’ expression in these women. Therefore, our results may be useful to improve the clinical management of women with BBD identified in the screening context since they are mostly nonproliferative diseases.

We found that women with an ER expression higher than 90% in BBD had double the risk of subsequent breast cancer compared with an ER expression lower than 70%. This finding is in agreement with the hypothesis that estrogens promote normal growth of breast epithelium but it can also have an important role in the pathogenesis of breast cancer. Our results are consistent with two previous studies that reported that ER expression in nonatypical hyperplasia is associated with an increased risk of breast cancer 8, 20. Other authors, however, have observed that ER expression does not increase the risk 7, 15. The type of included benign lesions can explain the differences with our results. Whereas we included nonproliferative and proliferative diseases, Huh et al. 7 assessed normal breast tissue selected from biopsies with confirmed BBDs and Barr et al. 15 included only atypical hyperplasia.

We observed that high PR expression in BBD was associated with increased risk of breast cancer. Increased PR expression has also been observed in hyperplastic enlarged lobular units that are considered as the earliest histologically identifiable lesion with premalignant potential 9. We did not find any other studies assessing PR expression in women with BBD. We found, however, one study that reported a decreasing trend for PR expression along with progression to malignancy 14. This finding seems contrary to our results. Therefore, we consider that the PR expression should be further evaluated in larger studies.

Regarding Ki67, we found a nonsignificant association between high expression and subsequent breast cancer. Conversely, previous studies reported significant associations in women with sclerosis adenosis 10 or during the first 10 years post atypical hyperplasia 11. In another study, Huh et al. 7 included women with BBD, mostly with proliferative disease, and observed a statistically significant increased risk of breast cancer in premenopausal women with >0.5% of KI67 expression in BBD. In our study, we did not find a statistically significant increased risk. Discrepancies with our results can be explained by the sample size of our study, characteristics of included women, or the method used for quantifying the Ki67 expression and the obtained cut‐offs. Although Huh et al. 7 obtained 0.28% as cut‐off from digitalized methods, in our study, the great majority of specimens were classified in the group of ≤1% because pathologist's microscopic assessment did not provide lower cut‐offs.

A better understanding of the characteristics of BBD identified in the context of screening will advance our clinical management of these increased risk lesions. Associations between genetic mutations and ER and PR expression were not addressed in our study. However, we think that further investigation should focus on ER and PR expression as biologically plausible biomarkers of breast cancer risk 21. On the other hand, clinical trials of selective estrogen receptor modulators and aromatase inhibitors have been proven to be effective at reducing breast cancer risk in women with atypical benign diseases 2, 22. In agreement with these trials, we attempted to provide more accurate risk estimates that may lead to the utilization of preventive treatment in women at high risk who are in a position to benefit from them. Furthermore, we think that a more intensive screening, with a shorter interval time between examinations, should be explored as a suitable modality for women with BBD and high ER or PR expression.

The main limitation of this study is the relatively small sample size. We were not able to analyze the subset of women with atypical lesions neither differences between in situ and invasive carcinomas because of the small sample size. Further studies with larger number of cases should focus on these women. In addition, the generalizability of our findings is limited to women with BBD who have undergone a biopsy with sufficient tissue to examine ER, PR, and Ki67 expression. On the other hand, this study is strengthened by the fact that two breast pathologists, who were blinded to later cancer outcomes, reviewed all samples. Reproducibility of pathologists’ microscopic examination compared with digitalized measures could be more useful for routine clinical practice in hospitals. Other strengths of this study are the use of a nested case–control design in the context of a screening program and the inclusion of nonproliferative diseases. Previous data came from studies assessing biologic characteristics of BBDs mostly identified in younger women and out of the context of population‐based screening programs. The current findings provide further evidence of the importance of nonproliferative diseases which are the most frequently detected BBDs in our setting 5.

In conclusion, a high expression of ER and PR in BBD is associated with increased risk of breast cancer. Although this increased risk was not demonstrated for the subset of nonproliferative or proliferative diseases, we found a tendency in both groups that should be confirmed in larger studies. Particularly in women with proliferative diseases, Ki67 may be associated with an increased risk. We believe that this information is helpful to better‐characterize BBD, and is one more step toward the possibility of personalizing the clinical management of these women in the screening context.

## Conflict of Interest

None declared.
